# Supplementary material for: Spatial domain detection using contrastive self-supervised learning for spatial multi-omics technologies
Source: Genome Res. 2025 Jul;35(7):1621–32. doi: 10.1101/gr.279380.124 (PMC12212350; doi:10.1101/gr.279380.124)
Supplement: Supplement 1 [file Supplemental_Material.pdf]

Supplemental Materials

Spatial domain detection using contrastive self-supervised learning for spatial multi-omics technologies

Jianing Yao, Jinglun Yu, Brian Caffo, Stephanie C. Page, Keri Martinowich, Stephanie C. Hicks*

*Correspondence to shicks19@jhu.edu

Contents

1. **Supplemental Notes S1-S5**
2. **Supplemental Figures S1-S18**

Supplemental Note S1: Limitations of the Silhouette Index for spatial transcriptomics data

To assess the degree of separation between clusters, we calculated the Silhouette index (Rousseeuw 1987) for Proust, GraphST, SpaGCN, and STAGATE using the CK-p25 mouse tissue dataset and human DLPFC Visium SPG dataset. We chose this metric because it does not depend on the use of a "gold standard" or "ground truth" (e.g., compared to Adjusted Rand index) and it is widely used in the field of single-cell transcriptomics to evaluate unsupervised clustering algorithms. We found that while Proust results in the highest Silhouette score compared to other existing algorithms in the human DLPFC Visium SPG dataset (**Supplemental Fig. S7B**), it achieves the lowest score in the mouse dataset (**Supplemental Fig. S7A**). However, we believe there are several important caveats to using this metric that limit its use here. First, the Silhouette index is not designed for multi-omics data, it is designed for one data modality so how to adapt it for multi-omics data is unclear. Secondly, the Silhouette index is not well-suited for spatial omics data, as it treats observations as independent and ignores the local spatial relationships between them. This means that the index does not account for the spatial context that is often critical in understanding biological patterns within spatial omics data. Additionally, STAGATE yields the highest Silhouette score while detecting regions with the least biological sense compared to other methods in the mouse dataset (**Supplemental Fig. S2**). In this way, we argue that the results we calculated using the Silhouette index are difficult to interpret and motivate an urgent need to develop alternative quantitative metrics to evaluate spatial multi-omics clustering algorithms.

Supplemental Note S2: Computation time and memory usage

To evaluate Proust's computational efficiency, we measured its processing time and peak GPU memory usage at each stage of the deep learning model. In each dataset, Proust exhibits variability in processing time, with the CNN image processing stage typically taking longer than the subsequent GNN-based stages (**Supplemental Fig. S16A**) due to the intensive computations required for extracting features from high-dimensional image data. Similarly, GPU memory usage is higher during image processing because of the large input sizes; however, it remains manageable for most applications. Although processing time and memory usage depend on the number of input channels, the performance remains within a practical range (**Supplemental Fig. S16B**). For instance, the CNN stage takes an average of 44.12 seconds for the Visium SPG DLPFC dataset with five channels, compared to 20.51 seconds for the Visium SPG mouse dataset with two channels. In contrast, the GNN Gene and GNN Image stages are less sensitive to changes in channel number.

Supplemental Fig. S16C compares the total computation time and peak GPU memory usage between Proust and GraphST for all samples. GraphST (mean time: 23.95 seconds; mean peak GPU: 521.97 MB) outperforms Proust (mean time: 101.91 seconds; mean peak GPU: 606.52 MB) primarily due to Proust's additional image preprocessing using CNNs and GNN-based feature extraction. Despite these extra steps, Proust's resource demands remain manageable, largely because a significant portion of the processing time is devoted to reading large raw .tiff image data. Overall, while Proust requires more resources than some alternative methods, its requirements are feasible for most research needs, especially in high-performance computing environments with job requests under 5 GB per sample.

Supplemental Note S3: Ablation study

We performed an ablation study comparing Proust against three baseline methods to evaluate its effectiveness: a simple PCA-based dimensionality reduction (first baseline), a GCN autoencoder without contrastive self-supervised learning (CSL) (second baseline), and a GCN autoencoder without the CNN preprocessing step (third baseline). All methods follow the same preprocessing steps for gene expression and image data as outlined in Sections Spatially-resolved gene expression preprocessing and Image feature extraction. The outputs are subsequently processed using the clustering steps described in Section Clustering and refinement. As shown in **Supplemental Fig. S17B**, Proust consistently achieves higher ARI scores across all samples in the Visium SPG DLPFC dataset, reflecting improved clustering performance relative to the baselines. **Supplemental Fig. S17A** further demonstrates that Proust produces spatially coherent clusters that better correspond with manual annotations and underlying tissue structures. Although the CNN preprocessing step may result in a minor loss of information, this loss is negligible because our primary focus is on capturing broad spatial patterns and protein distributions in IF images that complement the ST data. The lower ARI scores observed in the baseline underscore the value of the CNN step in retaining relevant information for spatial domain detection while reducing noise and processing time. Moreover, the incorporation of contrastive self-supervised learning aggregates neighboring information effectively, enhancing the representation of local spatial context and improving the delineation of adjacent clusters. Overall, these results highlight the benefit of incorporating graph-based CSL and CNN image preprocessing to refine the latent space representation for more accurate spatial domain discovery.

Supplemental Note S4: Adjustment of modality weights

The "weighting" in Proust refers to how many principal components (PCs) are used for each modality, which directly controls the relative contribution of gene expression and image data to the hybrid profile for clustering. By default, we selected the top 30 PCs for gene expression to retain the most significant features—capturing about 80% of the variance (**Supplemental Fig. S18A**), in line with common practice—and the top 5 PCs for image data, which account for over 90% of the variance and suffice to capture broad spatial patterns as supplementary information in this specific analysis. As shown in **Supplemental Fig. S18B**, reducing gene PCs to 5 causes a marked drop in ARI due to a considerable loss of nuanced information compared to the default setting. Additionally, ablation experiments using only gene PCs at various depths (**Supplemental Fig. S18C**) consistently underperform the full multimodal Proust framework, underscoring the value of integrating CNN-processed image features.

We also demonstrated Proust's flexibility in **Fig. 4D** by using the top 10 PCs from both reconstructed gene expression and reconstructed Aβ/pTau image features to up-weight pathology-related signals compared to the default setting. The results show that Proust can detect disease-associated spatial domains in addition to canonical cortical layers by adjusting the number of PCs for each data modality and highlight how Proust can be tailored to different biological contexts.

Lastly, we recognize the challenge of selecting appropriate weights between different data modalities, as this is a user-defined parameter in Proust. Users can adjust the weights assigned to gene expression and protein information depending on the amount of information they want each modality to contribute to the clustering results. However, they may find it challenging to determine the exact weights to assign to each modality, especially when there is no manual annotation or ground truth available for reference. To address this, Proust provides default values for the number of PCs used for each modality, which have shown to work well across a range of datasets, while also allowing users to adjust these settings according to their needs. We also offer the option for users to specify how much variance they wish to capture from each modality, and the algorithm will automatically determine the appropriate number of PCs accordingly.

Supplemental Note S5: Design choices and novelty in Proust

Choice of CNN-based autoencoder

Several recent spatial omics tools first learn compact image representations with CNN autoencoders before integrating them with other biological data types. For instance, STACI (Zhang et al. 2022) applies a variational CNN to chromatin images and then fuses those embeddings with transcript counts, ConGI (Zeng et al. 2023) uses a DenseNet121 backbone (pre-trained on ImageNet) to extract morphological features from histopathology patches, and Yang et al. (Yang et al. 2021) process multi-channel single-cell images through a CNN autoencoder to enable cross-modality translation. In Proust, we adopt a lightweight CNN that encodes each immunofluorescence channel at the spot level to reduce dimensionality, smooth out high-frequency pixel noise, and capture broad patterns of protein distribution. The aim is to highlight general changes in protein distribution, offering complementary insights alongside spatially-resolved gene expression data. The resulting per spot feature vectors concisely summarize image context and serve as inputs to our subsequent graph-based stages.

Mean squared error for reconstruction

In our model, we employ mean squared error (MSE) as the reconstruction loss in both the CNN and GNN autoencoders because it directly penalizes large deviations between the original and reconstructed inputs. By averaging squared differences across all feature dimensions, MSE ensures that high-intensity regions, where important protein signals reside, are faithfully reconstructed while still preserving overall structure. We normalize each channel's inputs via min–max that is consistent with the gene expression data to prevent extreme intensity values from dominating training, stabilize the scale of reconstruction errors, and enable the network to learn balanced representations across both bright and dim regions.

Nonlinear multimodal fusion vs. linear factor models

Among the various strategies for spatial transcriptomics analysis, linear latent factor methods (e.g., MEFISTO (Velten et al. 2022) and NSF (Townes and Engelhardt 2023)) decompose data into interpretable factors with Gaussian-process regularization, while graph-based deep learning frameworks learn hierarchical, nonlinear embeddings directly from processed features. For instance, MEFISTO and nonnegative spatial factorization (NSF) decompose data via linear combinations of latent factors, whereas Proust's core is a deep, nonlinear graph convolutional autoencoder paired with contrastive self-supervised learning.

This design lets us learn hierarchical, nonlinear mappings from spatial transcriptomics data and CNN-derived image features that can be combined into a hybrid profile so that spatially adjacent (or biologically similar) spots cluster tightly. Crucially, Proust uniquely integrates both embeddings from immunofluorescence (IF) or histological images and transcriptomic data: the CNN step distills broad protein-distribution patterns, and the GCN layers then refine these alongside gene expression by aggregating information from neighboring spots. This nonlinear, multimodal fusion enables Proust to perform a comprehensive analysis of intricate spatial domains using transcriptomic information and imaging-based protein channels, which linear GP- or NMF- based approaches and other deep learning methods do not inherently support.

Supplementary References

Rousseeuw PJ. 1987. Silhouettes: A graphical aid to the interpretation and validation of cluster analysis. *J Comput Appl Math* **20**: 53–65. doi: 10.1016/0377-0427(87)90125-7.

Townes FW, Engelhardt BE. 2023. Nonnegative spatial factorization applied to spatial genomics. *Nat Methods* **20**: 229–238. doi: 10.1038/s41592-022-01687-w.

Velten B, Braunger JM, Argelaguet R, Arnol D, Wirbel J, Bredikhin D, Zeller G, Stegle O. 2022. Identifying temporal and spatial patterns of variation from multimodal data using MEFISTO. *Nat Methods* **19**: 179–186. doi: 10.1038/s41592-021-01343-9.

Yang KD, Belyaeva A, Venkatachalapathy S, Damodaran K, Katcoff A, Radhakrishnan A, Shivashankar GV, Uhler C. 2021. Multi-domain translation between single-cell imaging and sequencing data using autoencoders. *Nat Commun* **12**: 31. doi: 10.1038/s41467-020-20249-2.

Zeng Y, Yin R, Luo M, Chen J, Pan Z, Lu Y, Yu W, Yang Y. 2023. Identifying spatial domain by adapting transcriptomics with histology through contrastive learning. *Brief Bioinform* **24**: bbad048. doi: 10.1093/bib/bbad048.

Zhang X, Wang X, Shivashankar GV, Uhler C. 2022. Graph-based autoencoder integrates spatial transcriptomics with chromatin images and identifies joint biomarkers for Alzheimer's disease. *Nat Commun* **13**: 7480. doi: 10.1038/s41467-022-35233-1.

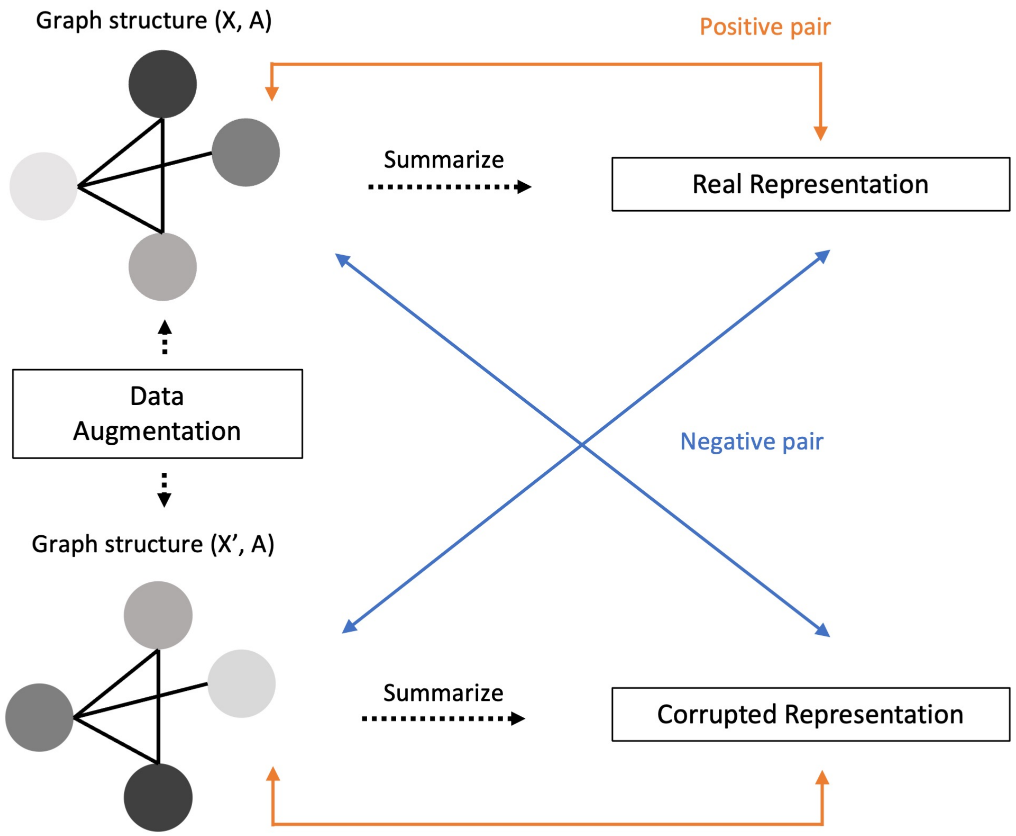

Figure S1: Contrastive self-supervised learning. Contrastive self-supervised learning is illustrated in this figure, demonstrating the refinement of latent representations during training of a graph-based autoencoder model. In the data augmentation step, biological features are randomly shuffled while preserving the distance-based graphs connecting each observation. Real and corrupted local representations are then summarized from these two sets of graph structures using a read-out function. A discriminative score for each pair of spot-patch representations is calculated during each iteration, comparing the spot-level latent embeddings with the summarized local context, respectively.

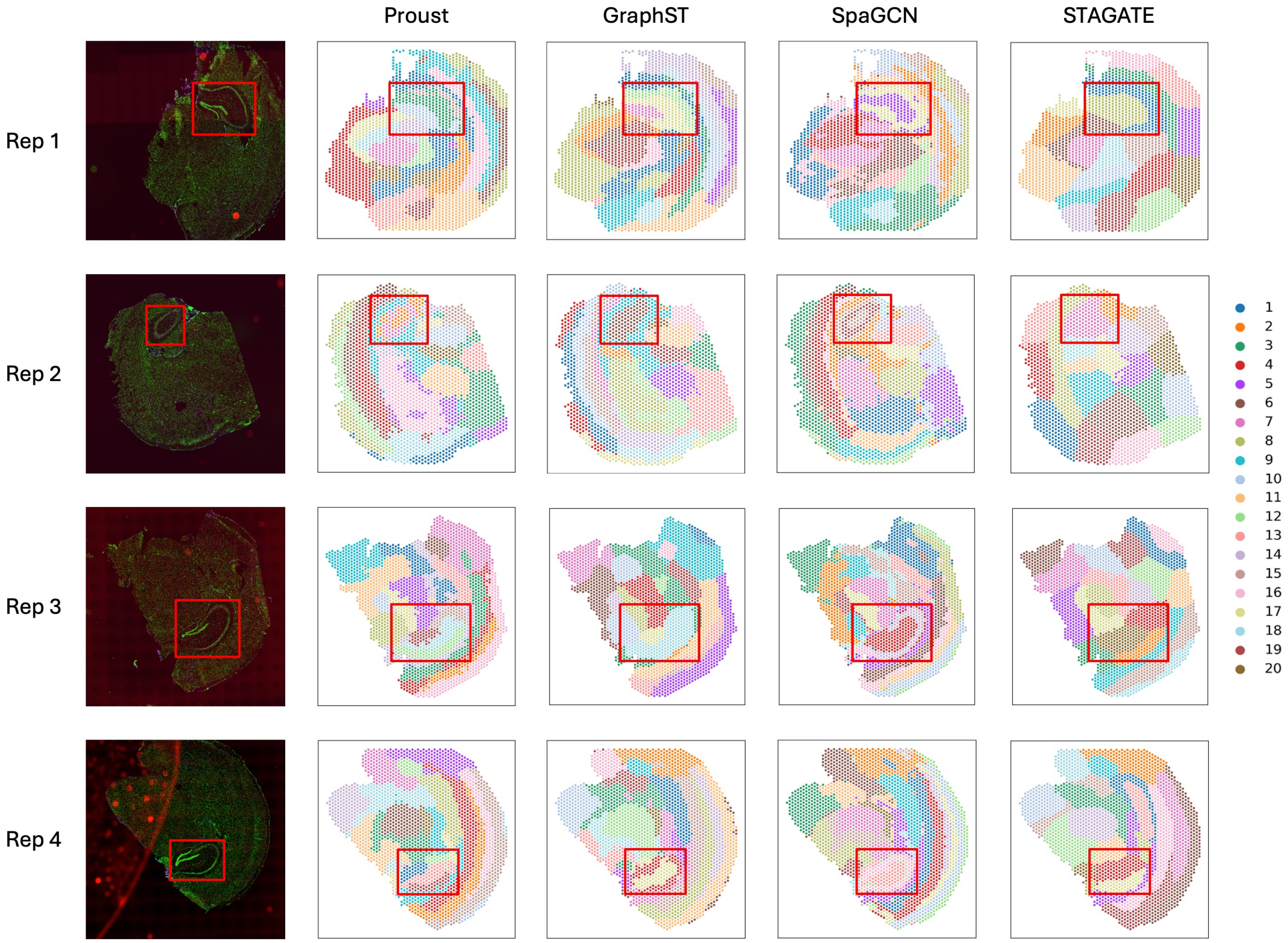

Figure S2: **IF staining images of γH2AX protein (first column) and the spatial domains detected by Proust, GraphST, SpaGCN, and STAGATE using cluster number k=20 on the Visium SPG CK-p25 mouse coronal brain tissue dataset.**

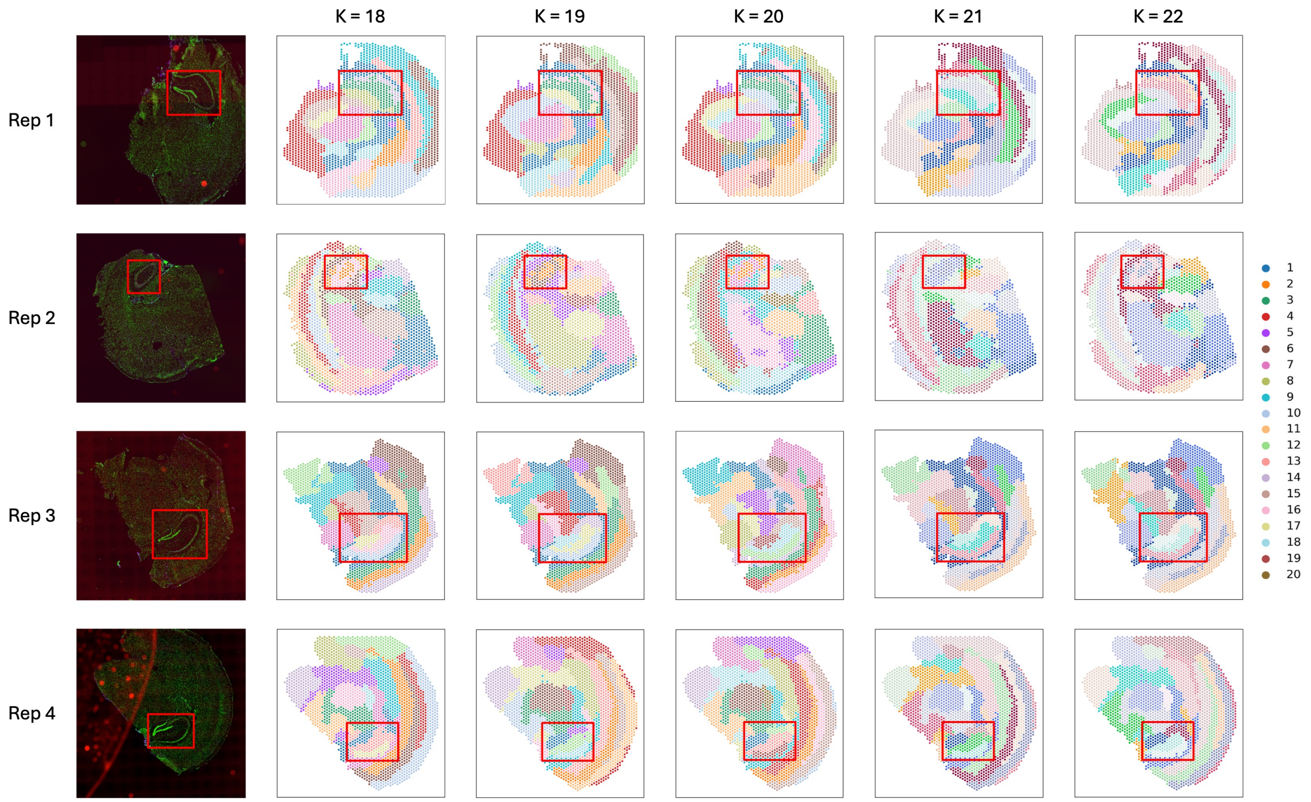

Figure S3: Clustering results of Proust on the Visium SPG CK-p25 mouse coronal brain tissue dataset using cluster number k = 18 - 22.

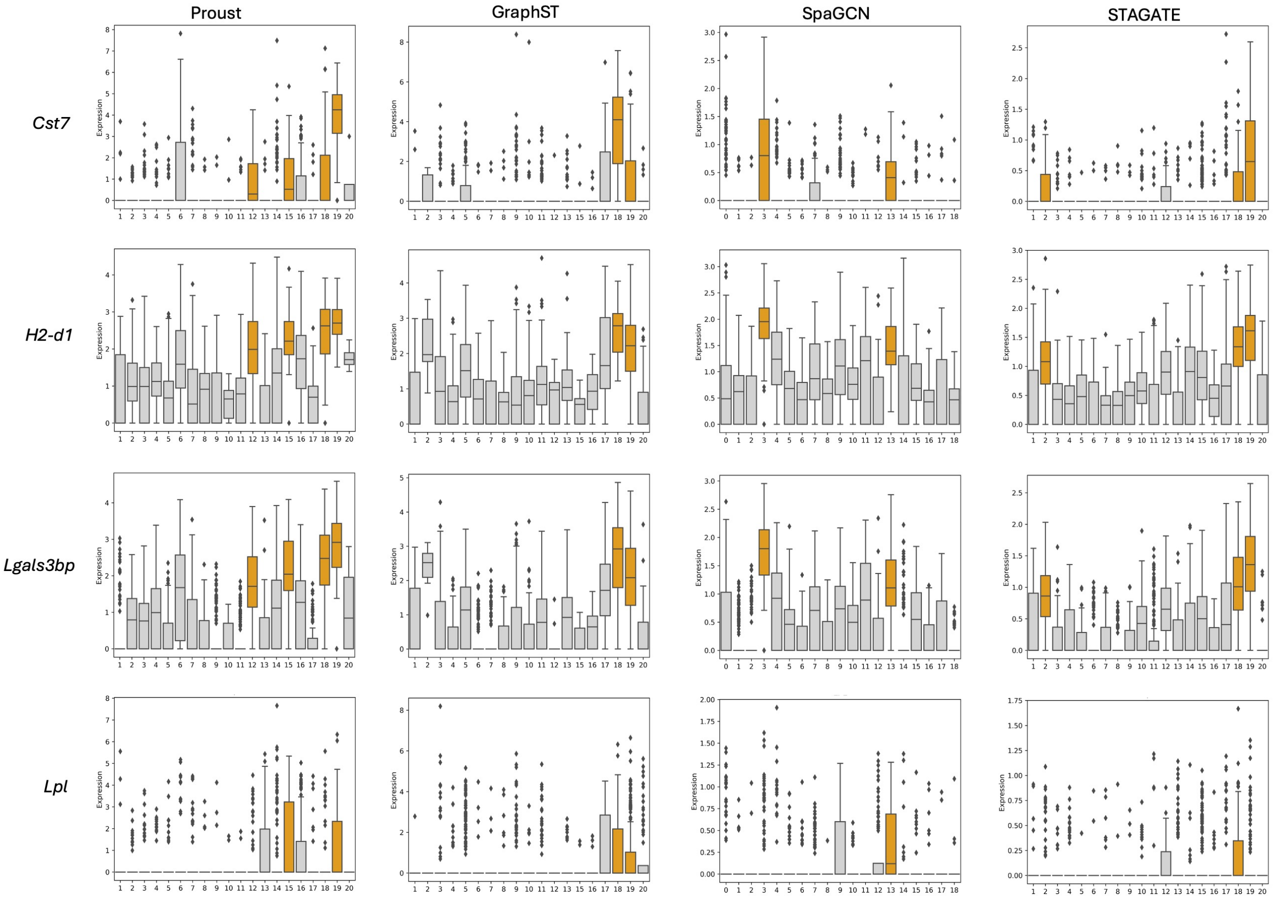

Figure S4: Expression level of reactive microglia marker genes on the Visium SPG CK-p25 mouse coronal brain tissue slide replicate 3. Boxplots of *Cst7*, *H2-d1*, *Lgals3bp*, and *Lpl* expression level grouped by clusters identified by Proust, GraphST, SpaGCN, and STAGATE. Hippocampus regions are depicted in orange; other regions are depicted in grey.

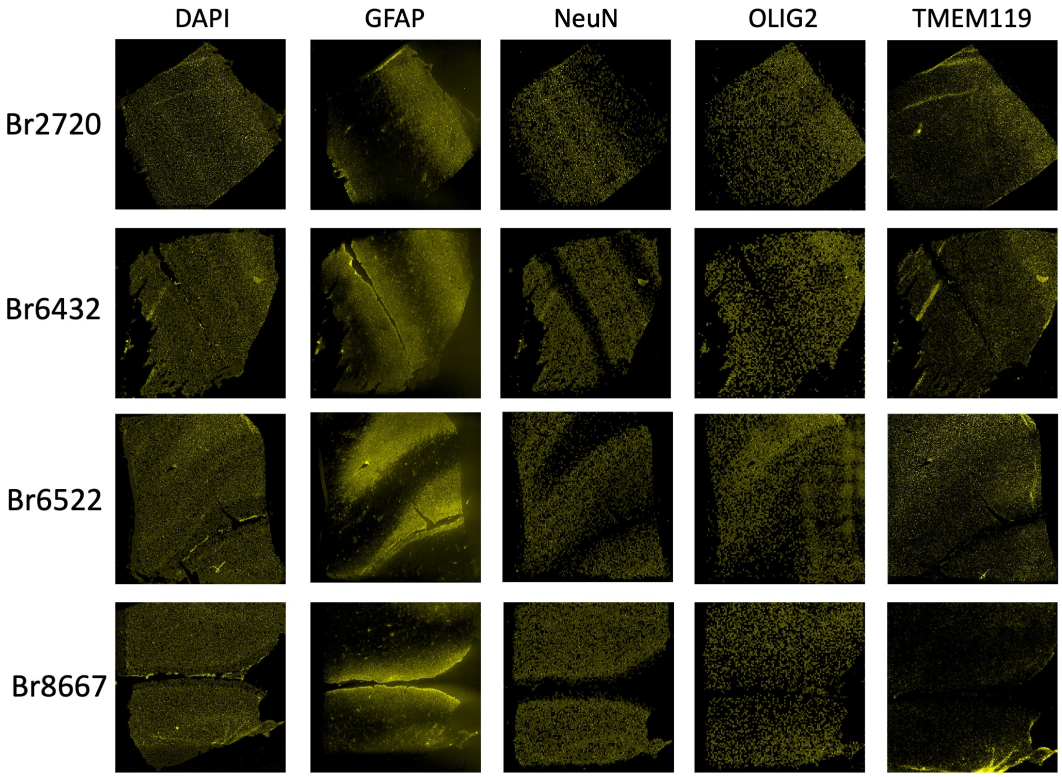

Figure S5: IF images of five cell-type channels (DAPI, GFAP, NeuN, OLIG2, and TMEM119) from four Visium SPG human DLPFC samples.

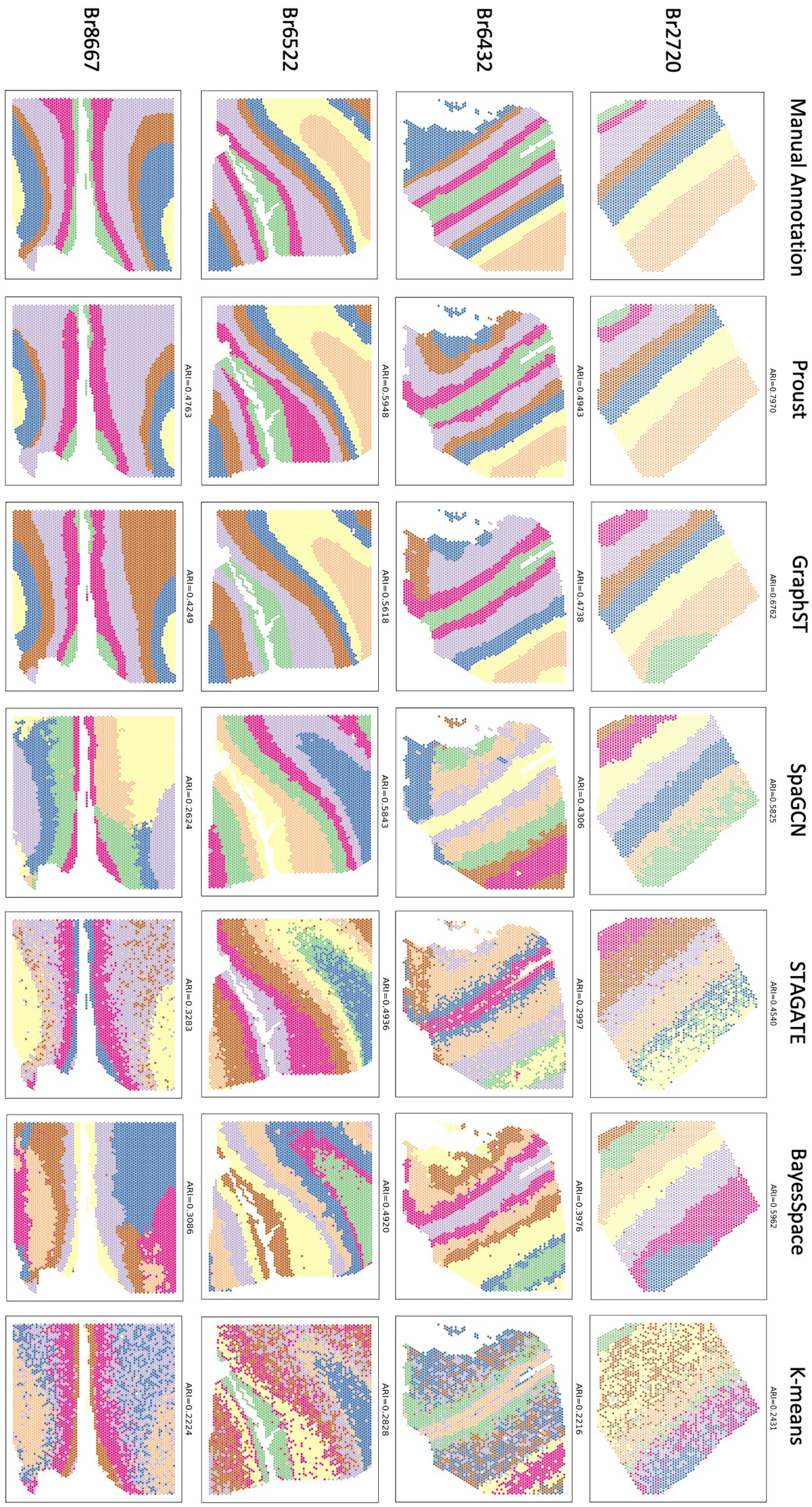

Figure S6: Manual annotations and clustering results from six methods on the Visium SPG human DLPFC samples.

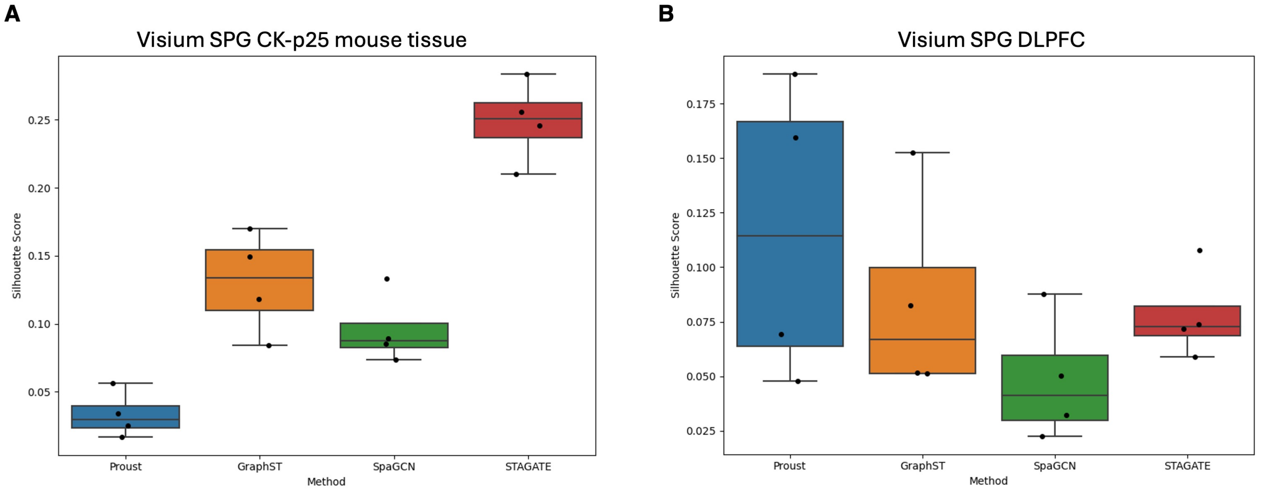

Figure S7: Silhouette scores of Visium SPG CK-p25 mouse tissue dataset and Visium SPG human DLPFC dataset. (A) Boxplot of Silhouette scores for Proust, GraphST, SpaGCN, and STAGATE on Visium SPG CK-p25 mouse tissue dataset. **(B)** Boxplot of Silhouette scores for Proust, GraphST, SpaGCN, and STAGATE on Visium SPG human DLPFC dataset.

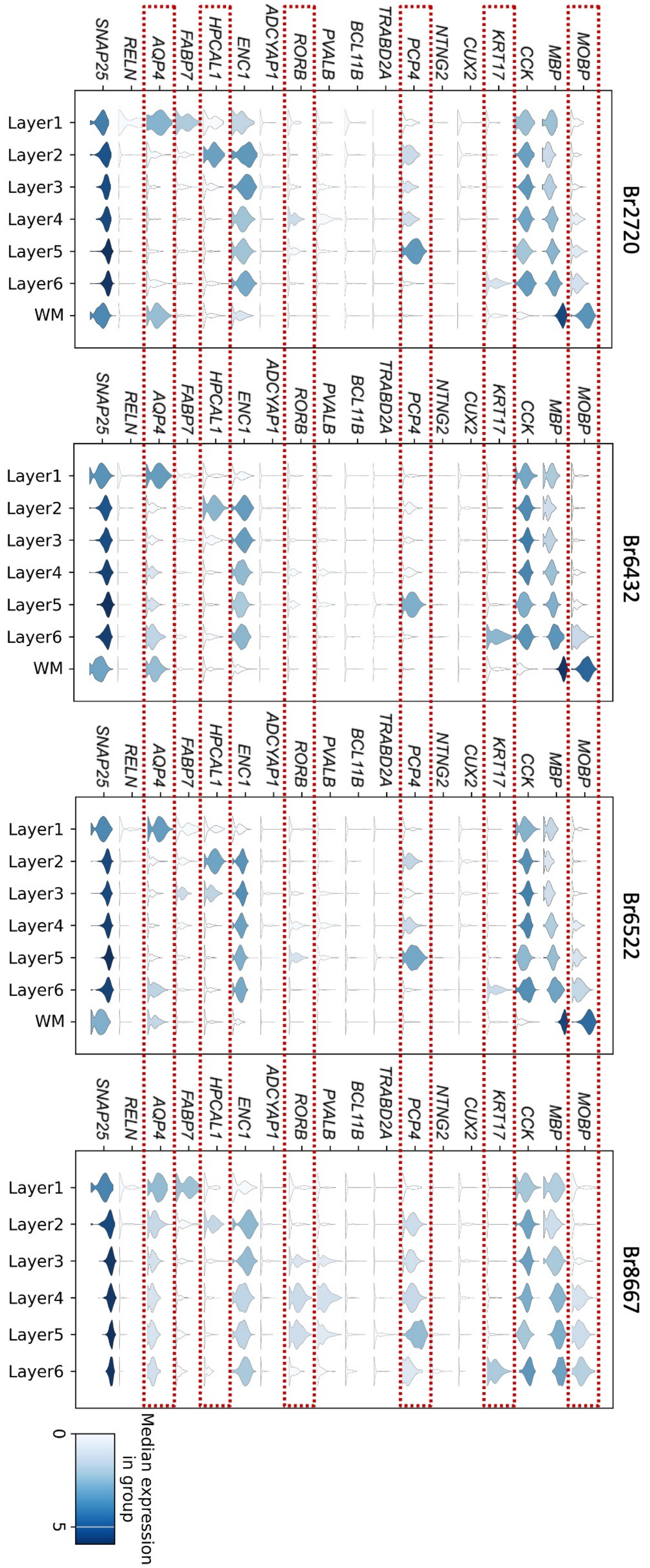

Figure S8: Stacked violin plots of known marker genes across clusters generated by Proust for four Visium SPG human DLPFC samples. Selected marker genes for each layer are boxed.

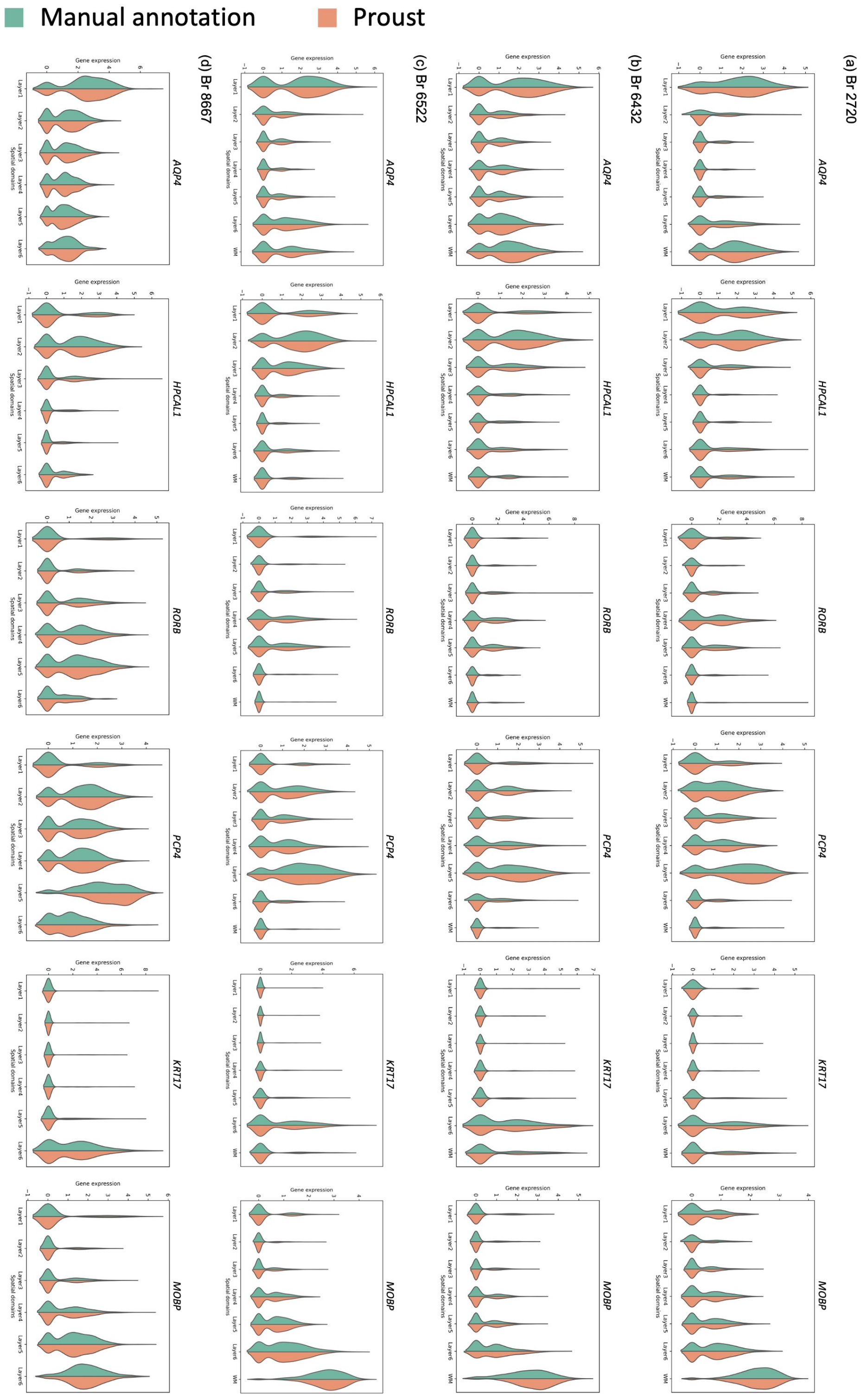

Figure S9: Violin plots to compare marker gene distributions within clusters identified by Proust and manual annotations of four Visium SPG human DLPFC samples.

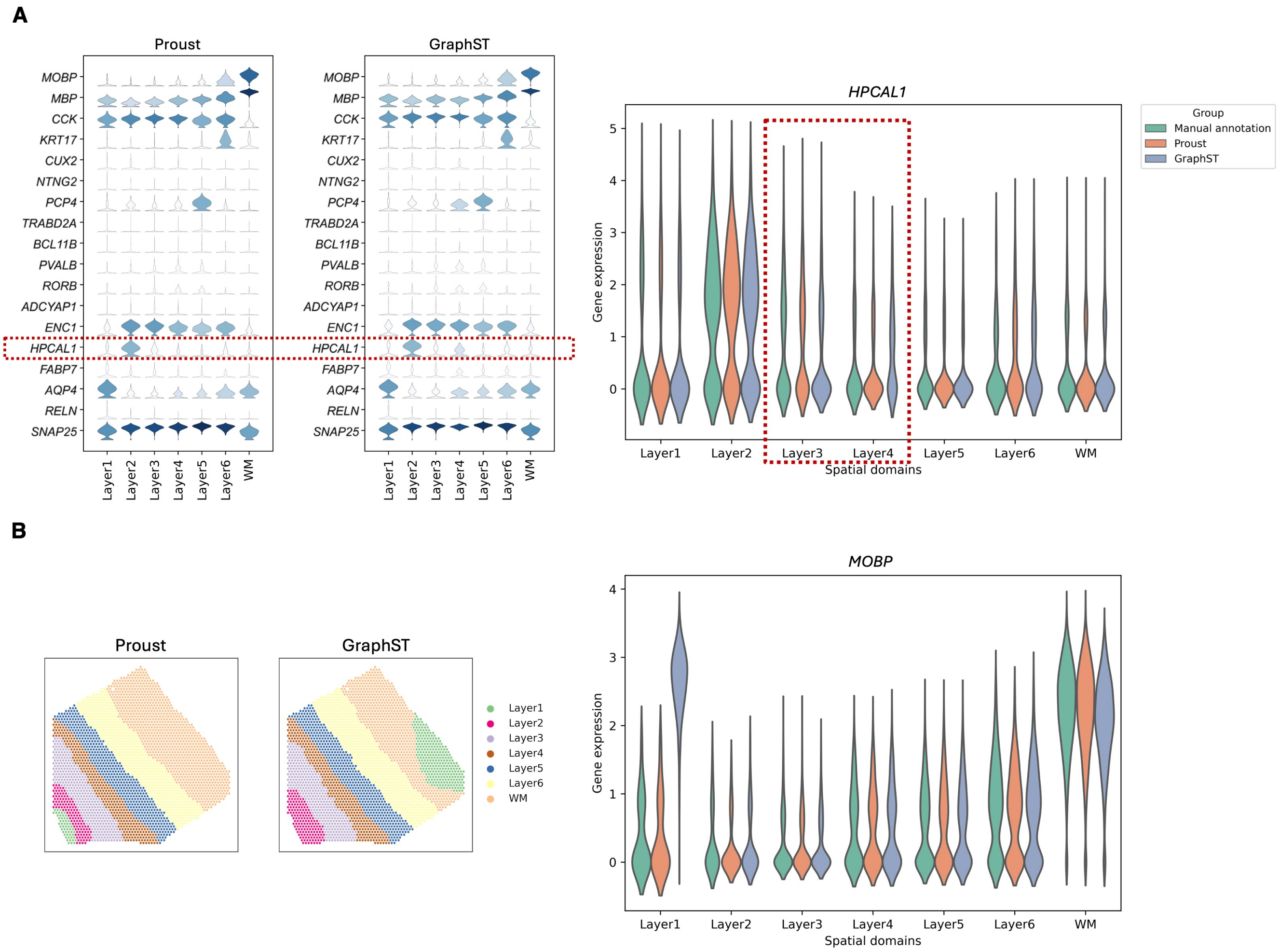

Figure S10: Comparison of marker gene expressions among manual annotation, Proust, and GraphST on Visium SPG human DLPFC samples. (A) Left: stacked violin plots of marker genes across clusters generated by Proust and GraphST for the Visium SPG human DLPFC Br6432 sample. Right: Violin plots to compare the distribution of *HPCAL1* within clusters identified by manual annotations, Proust, and GraphST of the same sample. **(B)** Left: clustering results from Proust and GraphST on the Visium SPG human DLPFC Br2720 sample. Right: Violin plots to compare the distribution of *MOBP* within clusters identified by manual annotations, Proust, and GraphST of the same sample.

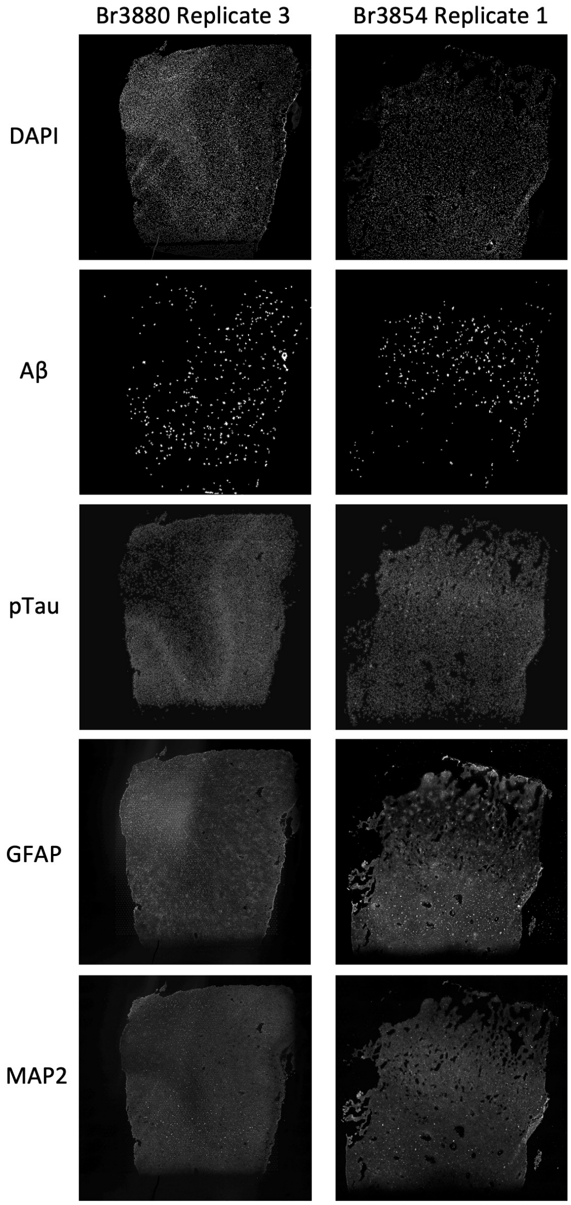

Figure S11: IF images of five protein channels (DAPI, Aβ, pTau, GFAP, and MAP2) from selected Visium SPG human inferior temporal cortex samples.

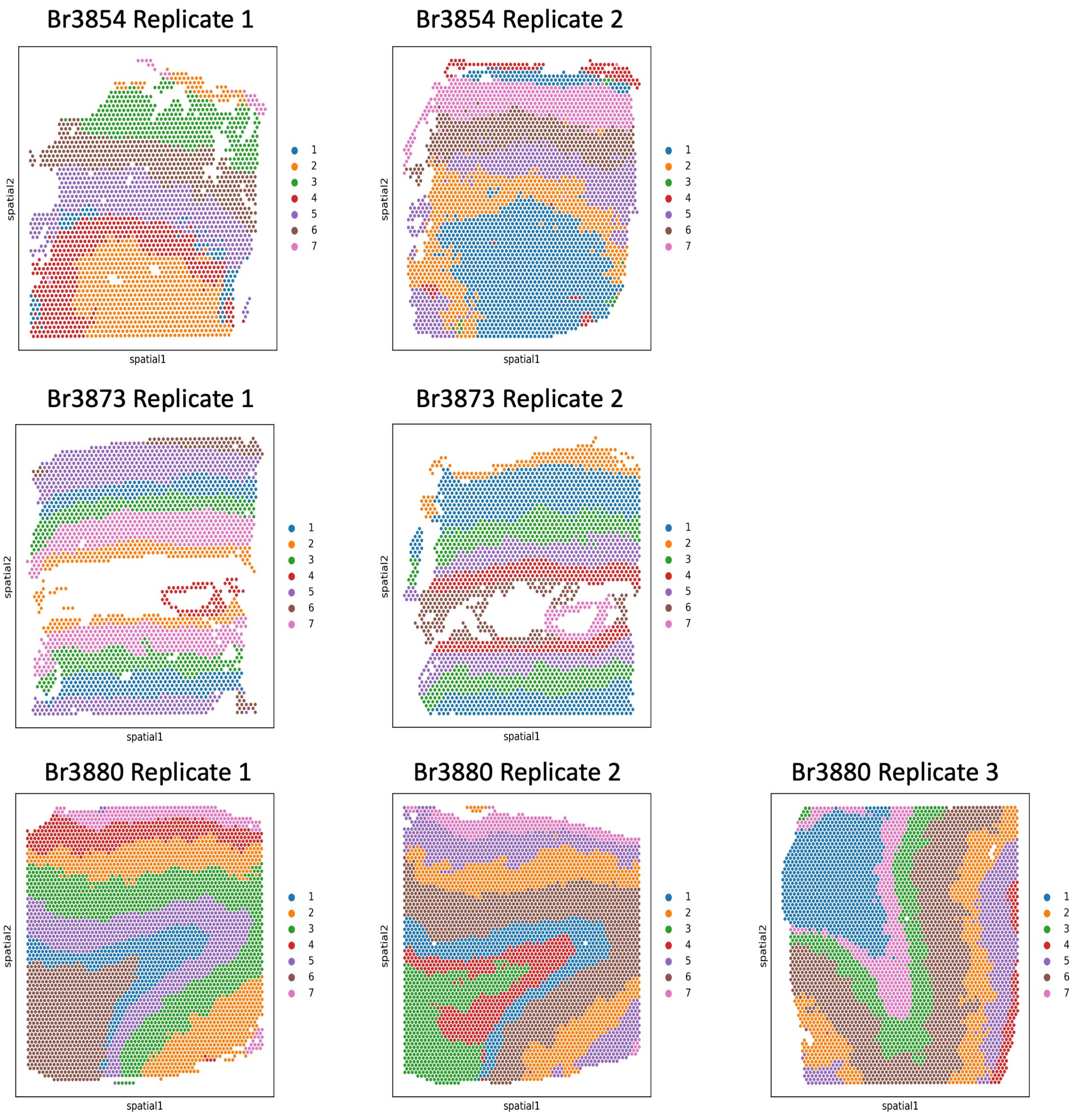

Figure S12: Proust clustering results of the Visium SPG human inferior temporal cortex samples, using five protein channels in Proust. Proust clustering results of seven Visium SPG human inferior temporal cortex samples, using five protein channels (DAPI, Aβ, pTau, MAP2, and GFAP), top 30 PCs from reconstructed gene expression, top 5 PCs from reconstructed extracted image features, and $k = 7$ clusters in Proust.

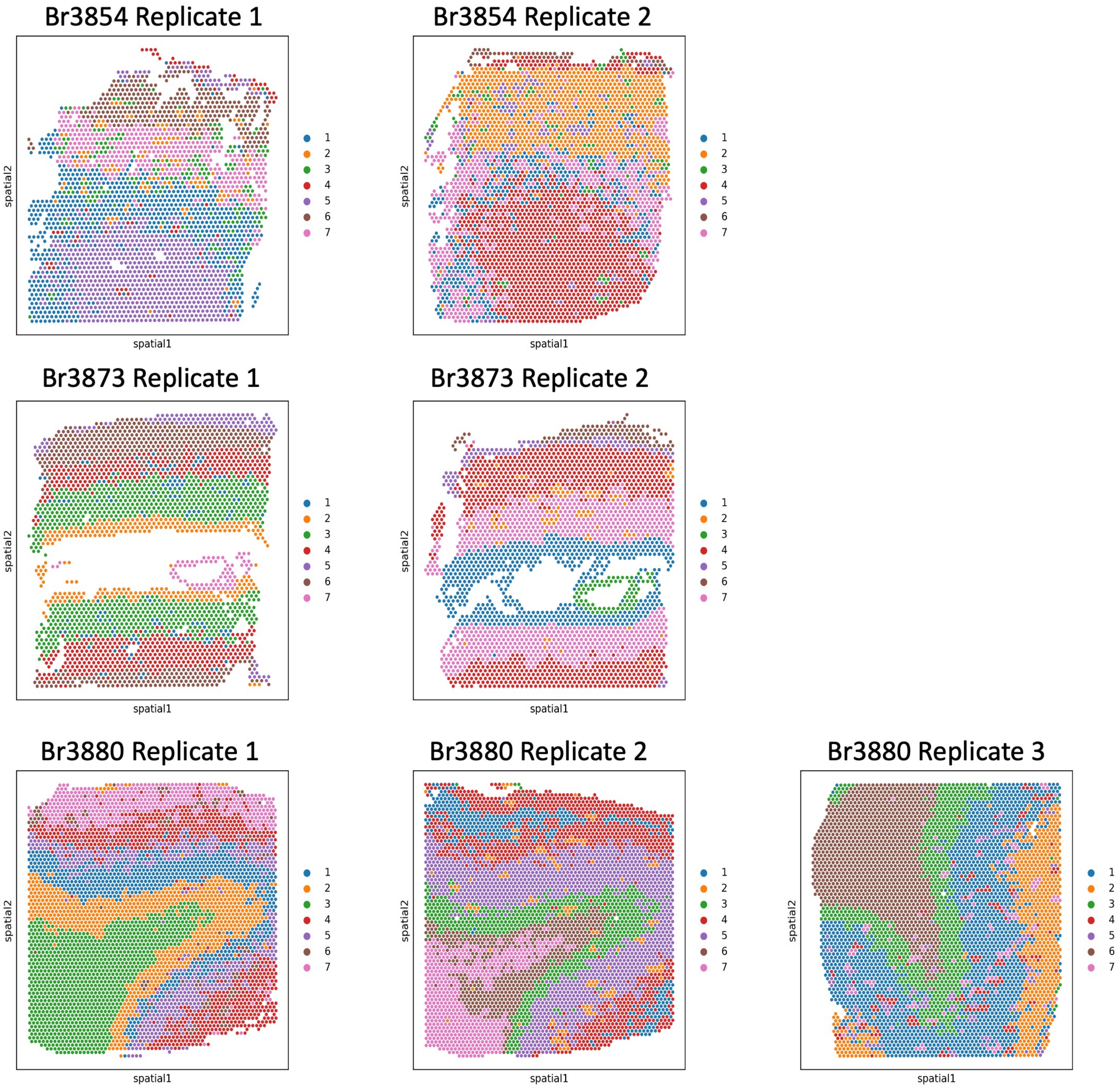

Figure S13: Proust clustering results of the Visium SPG human inferior temporal cortex samples, using two protein channels. Proust clustering results of seven Visium SPG human inferior temporal cortex samples, using two protein channels (Aβ and pTau), top 10 PCs from reconstructed gene expression, top 10 PCs from reconstructed extracted image features, and $k = 7$ clusters in Proust.

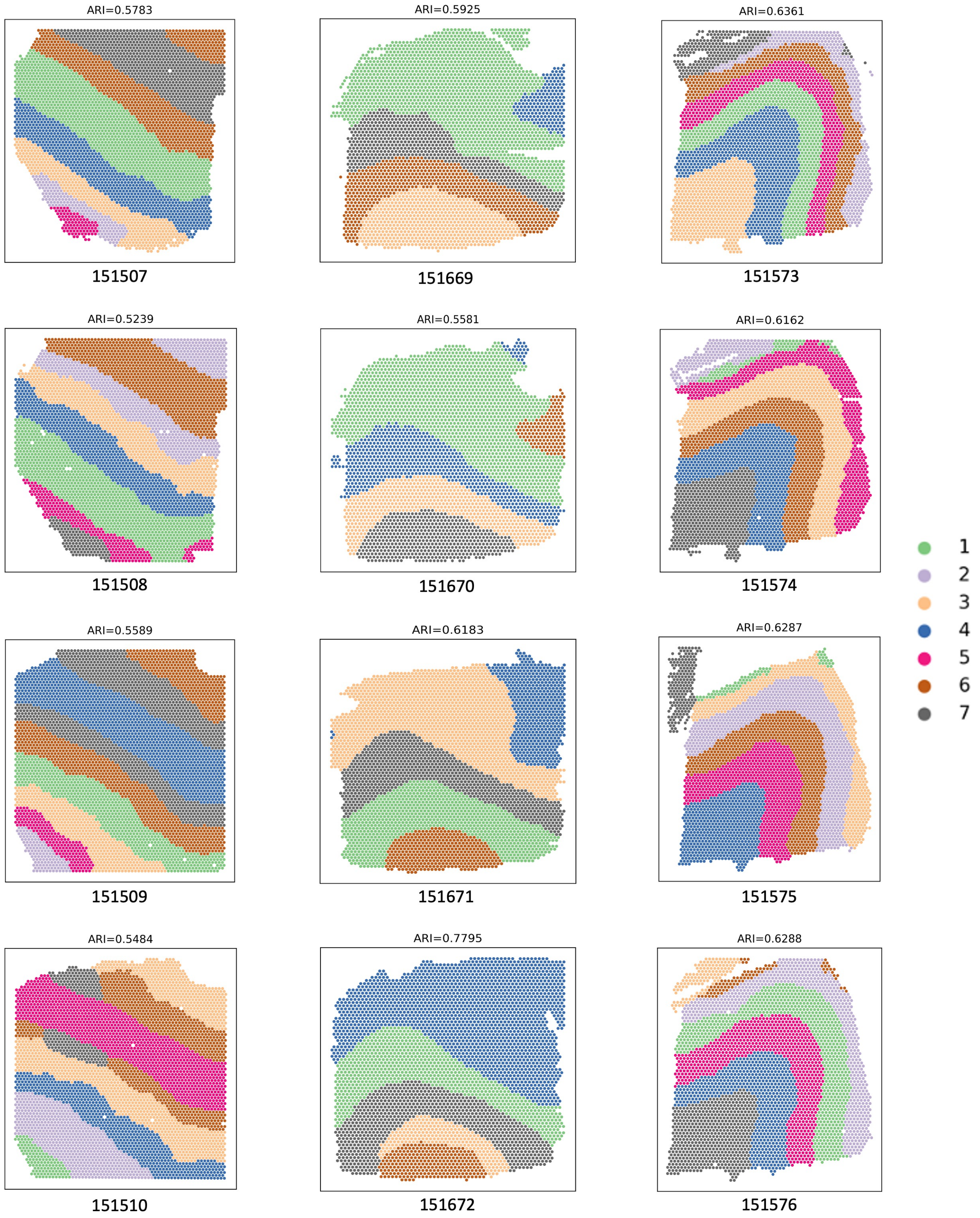

Figure S14: Proust clustering results of 12 Visium human DLPFC samples that contain H&E images.

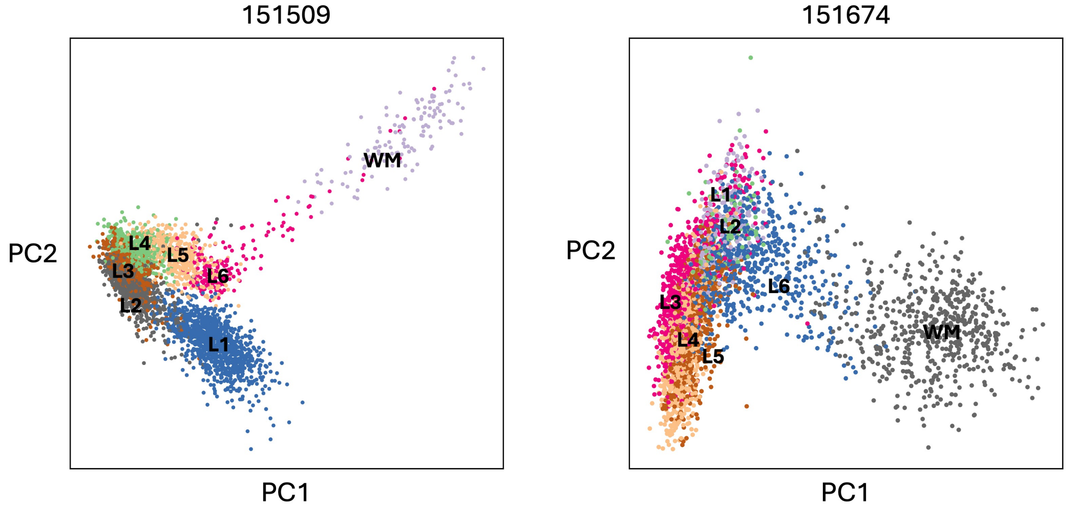

Figure S15: PCA plots of the first two principal components for sample 151509 and 151674 from the Visium human DLPFC dataset.

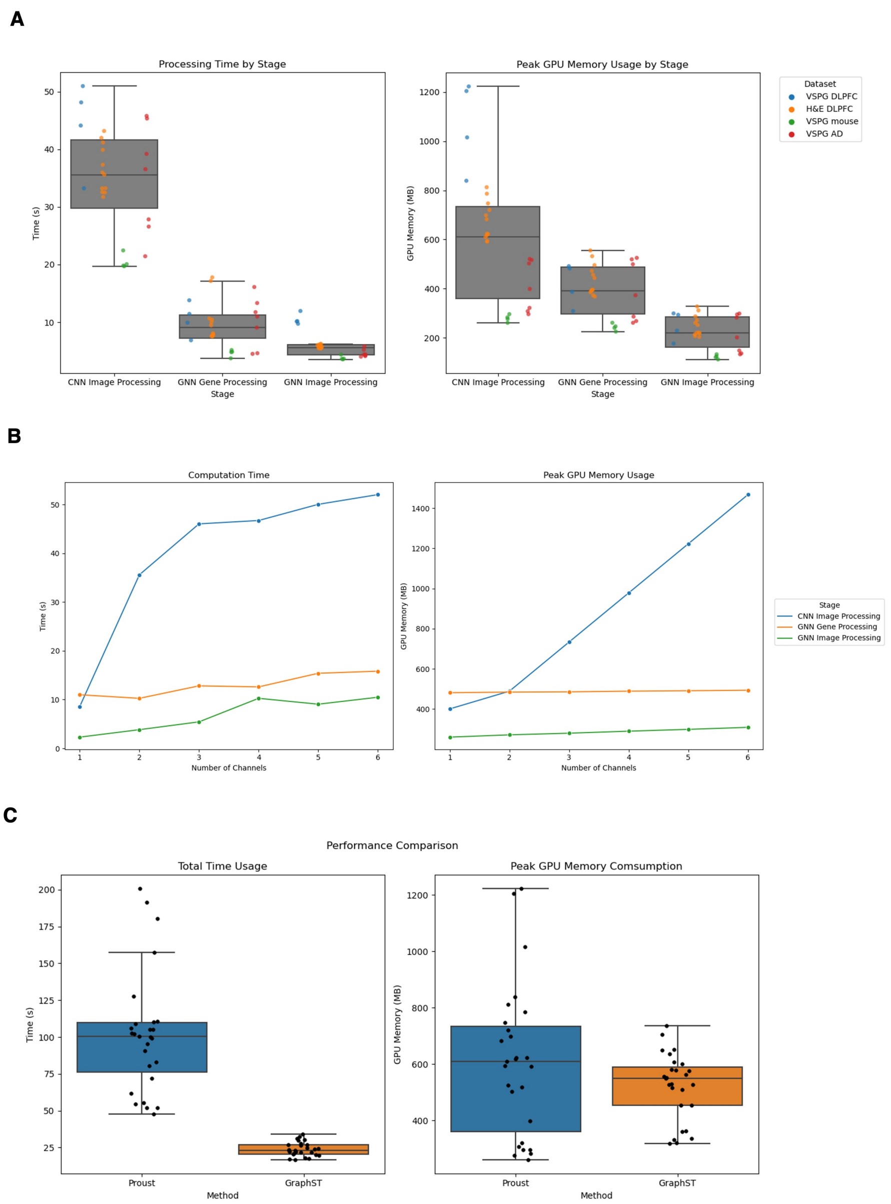

Figure S16: Boxplots of computation time and memory usage across datasets and competing methods. (A) Boxplots of computation time (in seconds) and peak GPU memory usage (in MB) for Proust at each stage of the deep learning process across four different datasets (VSPG DLPFC, H&E DLPFC, VSPG mouse, and VSPG AD). **(B)** Comparison of computation time and peak GPU memory usage across three stages of Proust as a function of the number of input channels (1 to 6) on sample Br6522 in the Visium SPG DLPFC dataset. **(C)** Comparison of total computation time and peak GPU memory usage between Proust and GraphST.

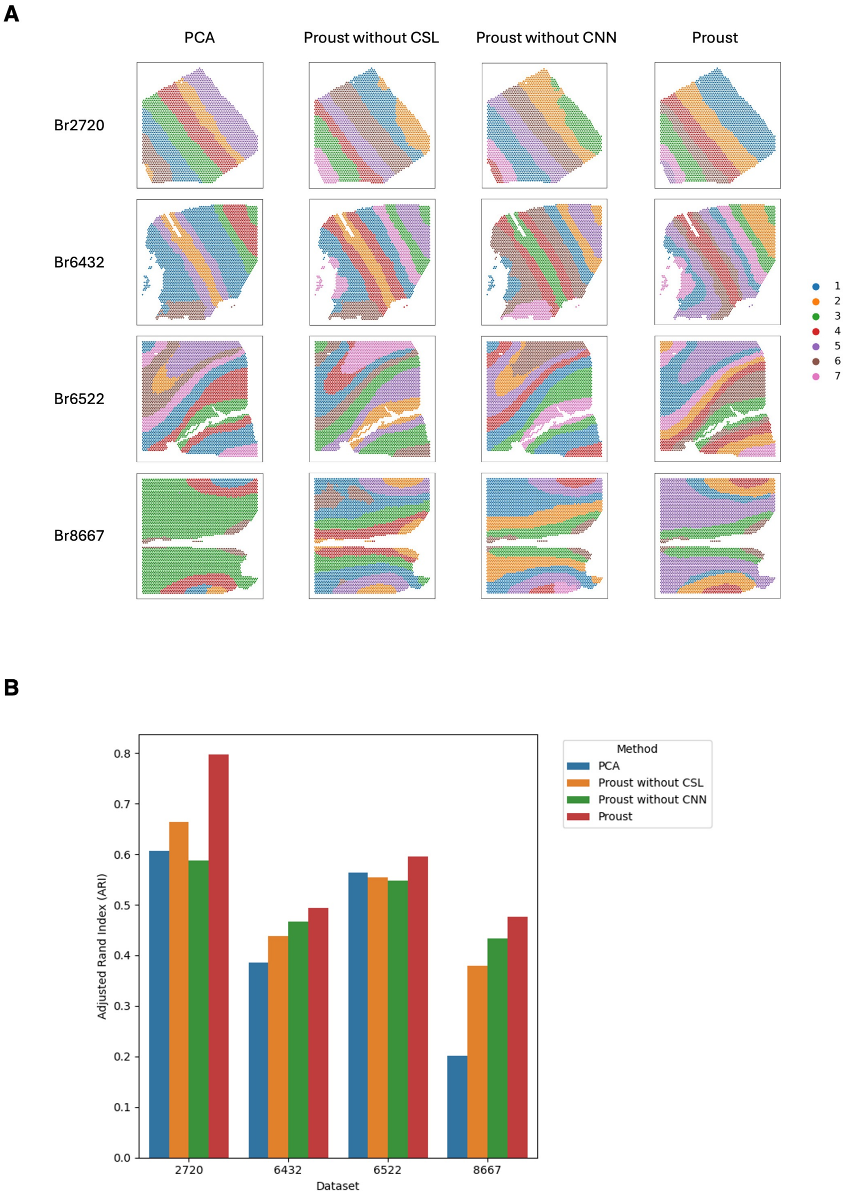

Figure S17: Comparison of Proust with three baseline methods (1) PCA-based dimensionality reduction and (2) Proust without contrastive self-supervised learning (3) Proust without CNN on the Visium SPG DLPFC dataset. **(A)** Spatial domain assigned by Proust and baseline methods. **(B)** Boxplot of clustering accuracy of Proust and three baselines based on adjusted rand index (ARI).

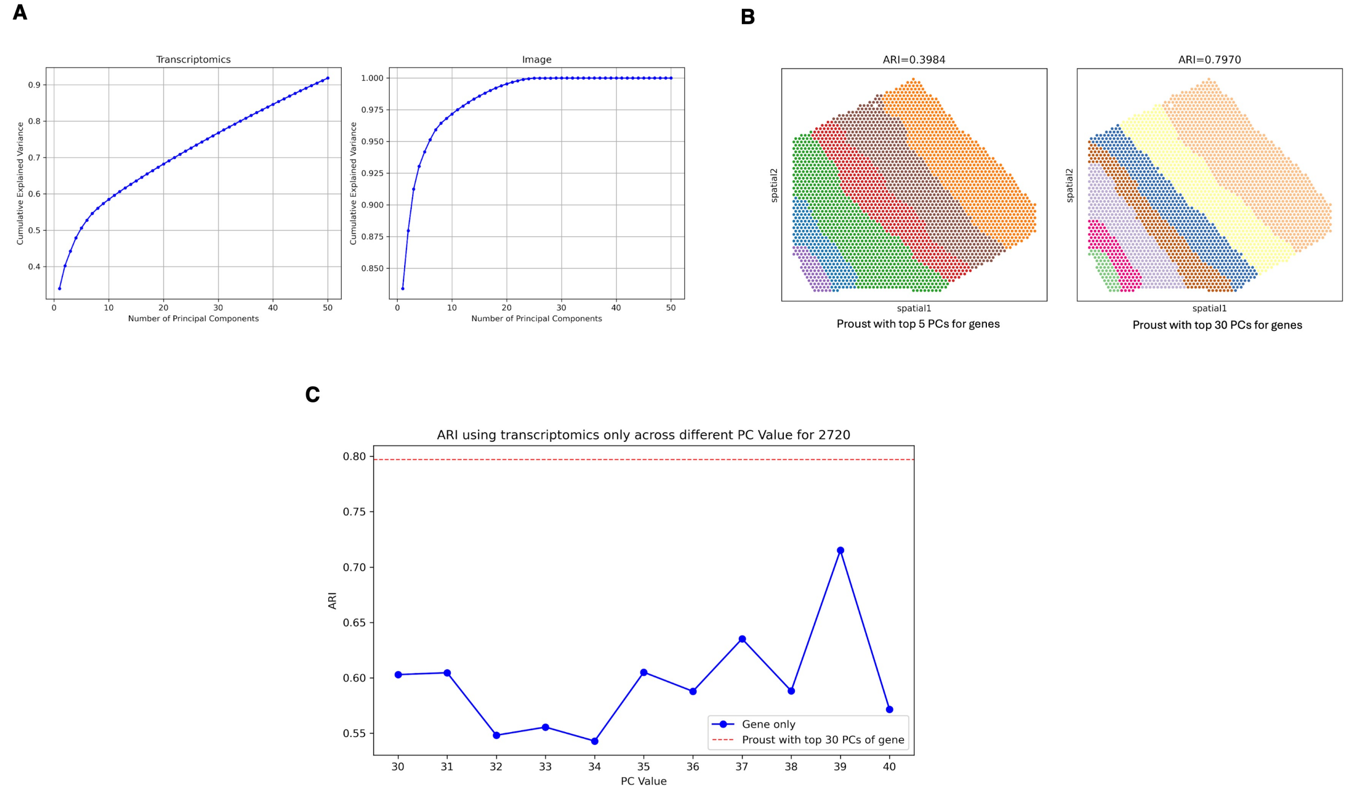

Figure S18: Analysis results with different modality weights on the Visium SPG DLPFC sample Br2720. (A) Cumulative explained variance by the number of principal components for transcriptomics and IF image reconstructed features. **(B)** ARI comparison with top 5 PCs vs top 30 PCs for transcriptomics in Proust. **(C)** ARI across different numbers of PCs for transcriptomic data using a gene-only model in Proust.
