# Supplementary figures and images for: Spatial domain detection using contrastive self-supervised learning for spatial multi-omics technologies
Source: Genome Res. 2025 Jul;35(7):1621–32. doi: 10.1101/gr.279380.124 (PMC12212350; doi:10.1101/gr.279380.124)

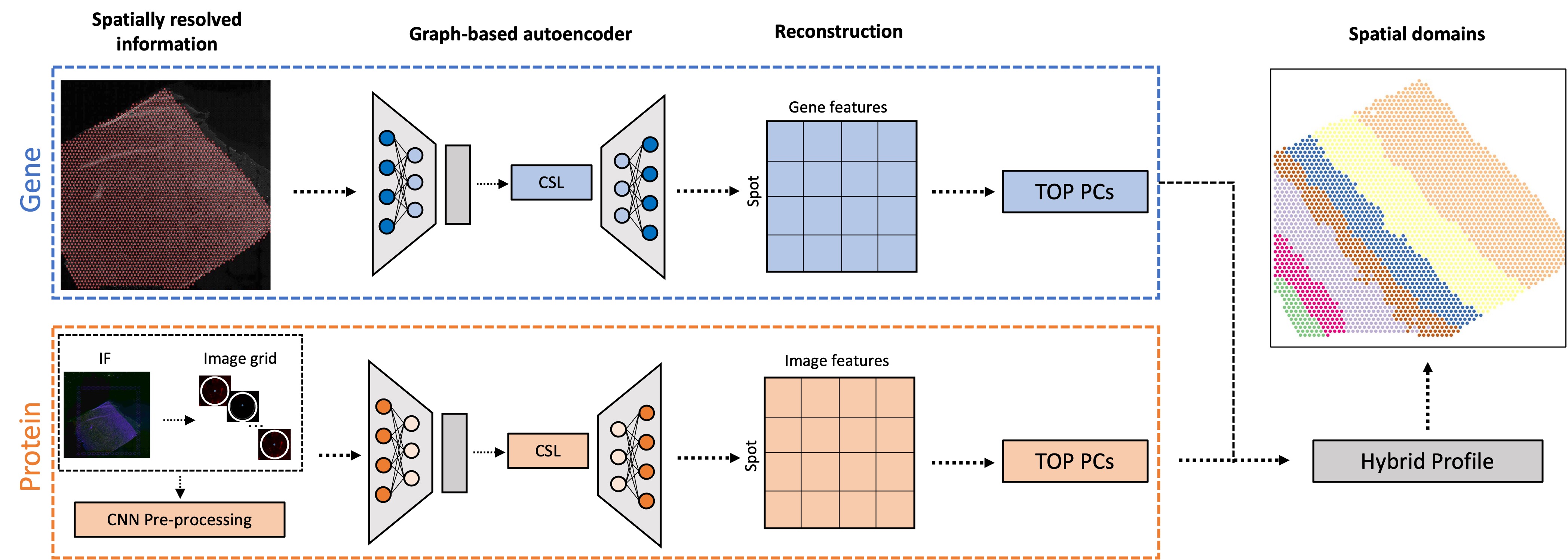

Supplement: Supplement 2 [file Supplemental_Code_.zip › Supplemental_Code/proust-master/Figure_1.jpg]
